# Correction: Huang et al. The Impact of Mutant *EDNRB* on the Two-End Black Coat Color Phenotype in Chinese Local Pigs. *Animals* 2025, *15*, 478

**DOI:** 10.3390/ani16213370

**Published:** 2026-10-26

**Authors:** Min Huang, Zuohao Wen, Tao Huang, Xiaolong Zhou, Zhijun Wang, Songbai Yang, Ayong Zhao

**Affiliations:** College of Animal Science and Technology · College of Veterinary Medicine, Zhejiang A&F University, Hangzhou 311300, China; minhuang0702@outlook.com (M.H.); wen824345107@outlook.com (Z.W.); taohuang@zafu.edu.cn (T.H.); zhouxiaolong@zafu.edu.cn (X.Z.); zhijunwang@zafu.edu.cn (Z.W.); sbyang@zafu.edu.cn (S.Y.)

Error in Figure

In the original publication [1], there was a mistake in Figure 5A as published. Figure 5A,D,E share the same gel band result; to eliminate any confusion, we have replaced Figure 5A. The objective of Figure 5A was to verify the presence of the target protein band, but not quantify protein expression. During the original preparation of Figure 5A, we used the same protein bands as those in the Co-IP experiments (Figure 5D,E) for clearer presentation. To avoid ambiguity, the revised Figure 5A was replaced by an independent protein expression experiment. The authors loaded 20 μg and 30 μg of protein in protein expression and Co-IP experiments, respectively. The authors used antibodies from ABclonal (ABclonal Biotechnology Co., Ltd., Wuhan, China) and Sigma (Merck KGaA, Darmstadt, Germany)/CST (Cell Signaling Technology, Danvers, MA, USA) in the protein expression and Co-IP experiments, respectively. These differences resulted in the difference in band intensity of the two experiments, but will not change the conclusions in the authors’ article. The corrected Figure 5 appears below.

In the original publication [1], Figure 1C was not cited. The citation has now been inserted in Section 3. Results, 3.1. Structural Prediction Showed That There Were Significant Differences in Three-Dimensional Structure Between Wild and Mutant EDNRB Proteins, Paragraph 1, and should read:

The results showed that the missing segment was 404–443 aa and was significantly different from the wild-type EDNRB protein (Figure 1C).

The authors state that the scientific conclusions are unaffected. This correction was approved by the Academic Editor. The original publication has also been updated.

## Figures and Tables

**Figure 5:**
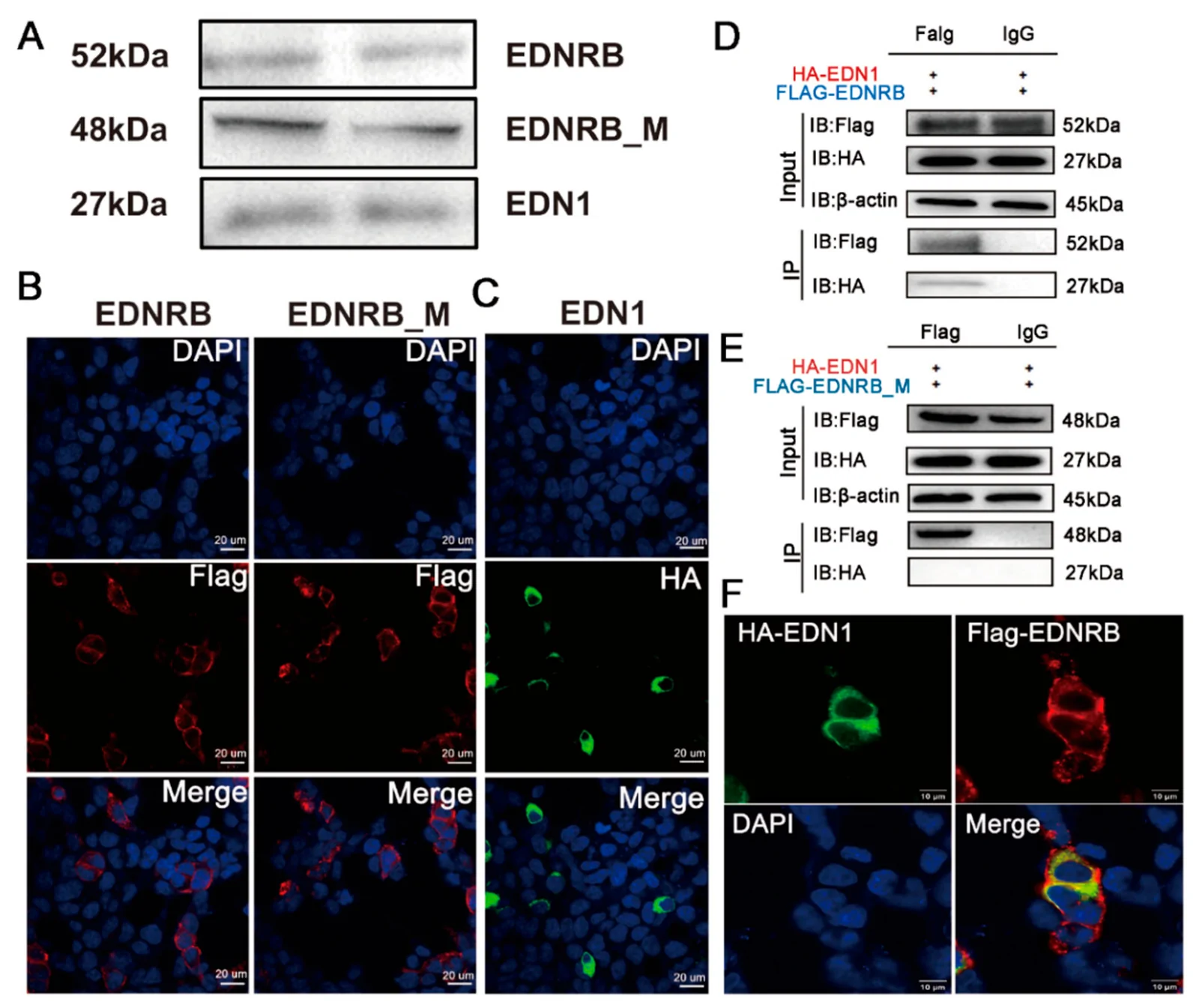
Recombinant plasmids were overexpressed; co-immunoprecipitation of wild and mutant EDNRB protein, and immunofluorescence of wild-EDNRB. (**A**) Protein expression after transfection of wild-*EDNRB*, mutant *EDNRB*, and *EDN1* into 293T cells. EDNRB: wild-type *EDNRB*; EDNRB_M: mutant *EDNRB*. (**B**) The immunofluorescence map of wild and mutant EDNRB. (**C**) The immunofluorescence map of *EDN1*. (**D**) The co-immunoprecipitation experiment between wild-EDNRB and EDN1 protein. (**E**) The co-immunoprecipitation between mutant EDNRB and EDN1 protein. (**F**) The immunofluorescence co-localization between wild-EDNRB and EDN1 protein.
