# Supplementary material for: The Impact of Mutant EDNRB on the Two-End Black Coat Color Phenotype in Chinese Local Pigs
Source: Animals (Basel). 2025 Feb 7;15(4):478. doi: 10.3390/ani15040478 (PMC11851453; doi:10.3390/ani15040478)
Supplement: Supplementary file 1 [file animals-15-00478-s001.zip › animals-3425682-supplementary/animals-3425682-supplementary.pdf]

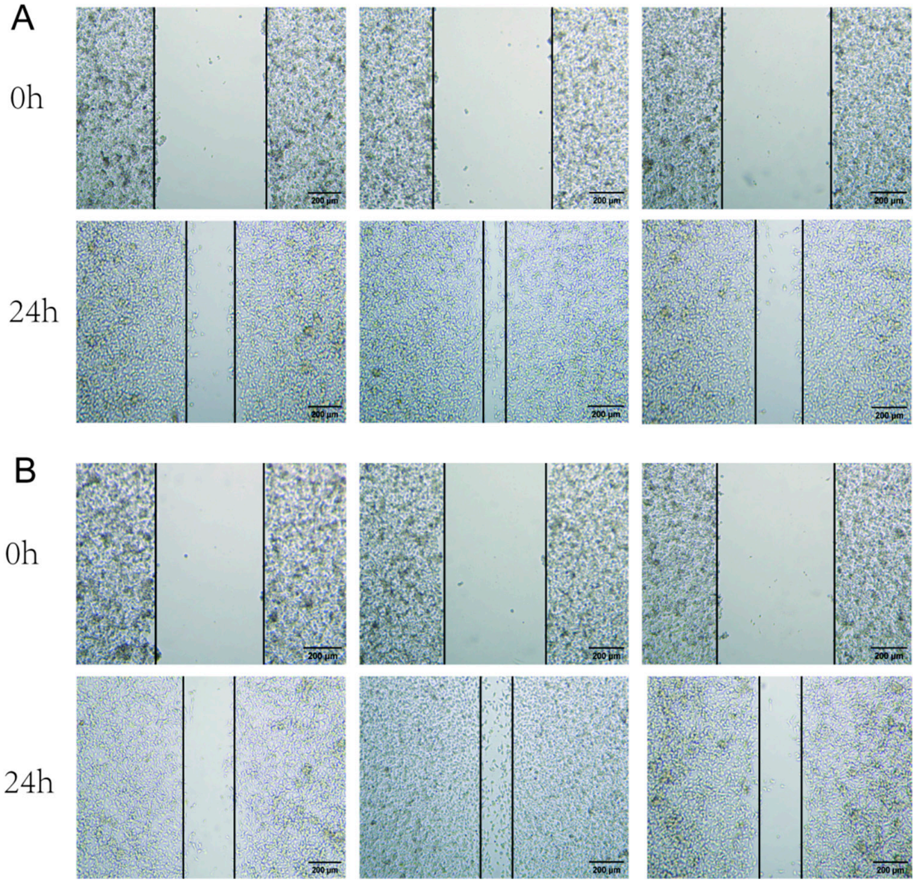

Figure S1 Cell migration map. (A) The second set of cell migration diagrams. (B) The third migration map of the cells.

Table S1 Wild EDNRB, mutant EDNRB, and EDN1 coding sequence

Gene coding sequence (5′→3′)	
Wild *EDNRB*	ATGCAGCCGCTGCGCAGCCTATGCGGACGCGCCCTG<br>GTGGCGCTGATCTTTGCCTGTGGCGTGGCCGGGGTC<br>CAGTCTGAGGAGAGGGGATTCCCGCCGGCCGGGGC<br>CACTCCACCAGCCCTGAGGACCGGAGAGATAGTGG<br>CGCCCCCTACTAAGACCTTCTGGCCCAGGGGGTCCA<br>ACGCCAGCCTGCCTCGGTCGTCCTCCCCCCCGCAGA<br>TGCCTAAAGGAGGGAGGATGGCGGGACCCCCAGCA<br>CGCACGCTCACCCCTCCTCCGTGCGAAGGACCCATC<br>GAGATCAAGGACACTTTCAAGTACATCAACACTGTG<br>GTGTCCTGCCTAGTGTTCGTGCTGGGCATCATCGGAA<br>ACTCCACACTGCTGCGAATCATTTACAAGAACAAGT<br>GCATGCGAAACGGCCCTAACATCTTGATAGCCAGCC<br>TGGCTCTGGGAGACCTGCTTCACATCATCATTGATAT<br>CCCCATCAACGTCTACAAGCTGCTCGCCGAGGACTG<br>GCCCTTTGGAGTTGAGATGTGTAAGCTGGTGCCTTTC<br>ATACAGAAGGCCTCCGTGGGAATCACTGTGCTGAGT<br>CTATGTGCTCTCAGTATTGACAGATATCGAGCCGTTG<br>CTTCTTGGAGTCGAATTAAAGGAATCGGGGTTCCAA<br>AATGGACAGCAGTGGAAATTGTTTTAATTTGGGTGGT<br>CTCCGTGGTCCTGGCCGTCCCGGAAGCCTTGGGTTTT<br>GACATGATTACCACTGACTATAAAGGAAATCGCCTG<br>CGAATCTGCTTGCTCCATCCTACTCAGAAAACAGCC<br>TTCATGCAGTTTTACAAGACAGCTAAAGATTGGTGG

	CTATTCAGTTTCTATTTCTGCTTGCCACTAGCCATCAC<br>TGCATTTTTTTATACCCTGATGACCTGTGAAATGCTGA<br>GAAAGAAGAGTGGCATGCAAATTGCTTTAAATGATC<br>ACTTAAAGCAGAGACGGGAAGTGGCCAAAACCGTA<br>TTTTGCCTGGTCCTTGTCTTTGCCCTGTGTTGGCTTCC<br>CCTTCATCTCAGCAGGATTTTGAAGCTCACTCTGTAT<br>GATCAAAATGATTCGAATAGATGTGAACTTTTGAGCT<br>TTTTGTTGGTATTGGATTACATTGGCATCAACATGGCG<br>TCCCTGAATTCCTGTATTAATCCAATAGCTCTGTATTT<br>GGTGAGCAAAAGATTCAAAAACTGCTTTAAGTCATG<br>CTTATGCTGCTGGTGCCAGTCATTTGAAGAAAAACA<br>GTCCTTGGAGGAAAAGCAGTCATGCTTAAAGTTCAA<br>AGCTAATGATCACGGATATGACAACTTCCGTTCCAGT<br>AATAAATACAGCTCATCTTGA
Mutant *EDNRB*	ATGCAGCCGCTGCGCAGCCTATGCGGACGCGCCCTG<br>GTGGCGCTGATCTTTGCCTGTGGCGTGGCCGGGGTC<br>CAGTCTGAGGAGAGGGGATTCCCGCCGGCCGGGGC<br>CACTCCACCAGCCCTGAGGACCGGAGAGATAGTGG<br>CGCCCCCTACTAAGACCTTCTGGCCCAGGGGGTCCA<br>ACGCCAGCCTGCCTCGGTCGTCCTCCCCCCCGCAGA<br>TGCCTAAAGGAGGGAGGATGGCGGGACCCCCAGCA<br>CGCACGCTCACCCCTCCTCCGTGCGAAGGACCCATC<br>GAGATCAAGGACACTTTCAAGTACATCAACACTGTG<br>GTGTCCTGCCTAGTGTTCGTGCTGGGCATCATCGGAA<br>ACTCCACACTGCTGCGAATCATTTACAAGAACAAGT<br>GCATGCGAAACGGCCCTAACATCTTGATAGCCAGCC<br>TGGCTCTGGGAGACCTGCTTCACATCATCATTGATAT<br>CCCCATCAACGTCTACAAGCTGCTCGCCGAGGACTG<br>GCCCTTTGGAGTTGAGATGTGTAAGCTGGTGCCTTTC<br>ATACAGAAGGCCTCCGTGGGAATCACTGTGCTGAGT<br>CTATGTGCTCTCAGTATTGACAGATATCGAGCCGTTG<br>CTTCTTGGAGTCGAATTAAAGGAATCGGGGTTCCAA<br>AATGGACAGCAGTGGAAATTGTTTTAATTTGGGTGGT<br>CTCCGTGGTCCTGGCCGTCCCGGAAGCCTTGGGTTTT<br>GACATGATTACCACTGACTATAAAGGAAATCGCCTG<br>CGAATCTGCTTGCTCCATCCTACTCAGAAAACAGCC<br>TTCATGCAGTTTTACAAGACAGCTAAAGATTGGTGG<br>CTATTCAGTTTCTATTTCTGCTTGCCACTAGCCATCAC<br>TGCATTTTTTTATACCCTGATGACCTGTGAAATGCTGA<br>GAAAGAAGAGTGGCATGCAAATTGCTTTAAATGATC<br>ACTTAAAGCAGAGACGGGAAGTGGCCAAAACCGTA<br>TTTTGCCTGGTCCTTGTCTTTGCCCTGTGTTGGCTTCC<br>CCTTCATCTCAGCAGGATTTTGAAGCTCACTCTGTAT<br>GATCAAAATGATTCGAATAGATGTGAACTTTTGAGCT<br>TTTTGTTGGTATTGGATTACATTGGCATCAACATGGCG<br>TCCCTGAATTCCTGTATTAATCCAATAGCTCTGTATTT<br>GGTGAGCAAAAGATTCAAAAACTGCTTTAAGTCATG<br>CTTATGCTGCTGGTGCCAGTCATTTGAAGAAAAACA<br>GTCCTTGGAGGAAAAGCAGTCATGCTTAAAGTTCAA<br>AGCTAATGATCACGGATATGACAACTTCCGTTCCAGT<br>AATAAATACAGCTCATCTTGA
EDN1	ATGGATTATTTCCCCATGATTATCGCTCTGCTGTTTGT<br>GGCTTTCCAAGGAGCTCCAGAAACAGCGGTCCTGG<br>GCGCCGAGCTCAGCCCGGGAGCCGAGAGCCAAGGG

	GAGACGCCCTCTCCCCATGCATCCTGGAGGCCCCGT CGGTCCAAGCGCTGCTCCTGCTCTTCCCTGATGGATA AAGAGTGTGTCTACTTCTGCCACCTGGACATCATCTG GGTCAACACTCCAGAACACATTGTCCCATACGGACT TGGAAGCCCTTCTAGGTCCAGGCGATCCTTAAAGGA TTTGTTTCCTGCAAAGGCAGCAGACCGCAGGGATAG ATGCCAGTGTGCCAGCCAAAAAGACAAGAAGTGCT GGAGTTTCTGCCAAGCAGGAAAAGAAATCGGCAGG GACCAAGACACAATGGAGAAACGCTGGGATAACCA AAAGAAAGGAACAGACTGTTCCAAGCTTGGAGAGA AGTGTATTCATCGGCAGCTGGTGATGGGAAGAAAAA TAAGAAGGTTGGAGGCCATCAGCAACAGCATCAAA ACATCTTTTCACATCGCCAAGCTGAAAGCCGAGCTC TACAGAGATAAGAAAGTGACCCATAACCGAACACA CTGA

Table S2 Primers used for RT-PCR

**Primer names**	**Sequences (5′→3′)**
GAPDH	Forward: GTGAAGGTCGGTGTGAACGGATT<br>Reverse: GGTCTCGCTCCTGGAAGATGGT
PLCγ	Forward: TCTGGCGGAATGGGAAAGTC<br>Reverse: TTCGTCTGTGGAACAGGCTC
Raf	Forward: CGTGGAGACGAGTGTCGAG<br>Reverse: CAAGTCCGCTCGGCTCTAAC
MITF	Forward: CCCCCATCTTTCTCAGGTGC<br>Reverse: ACGATTCCCGATTCGGACTG
