# Supplementary figures and images for: The Impact of Mutant EDNRB on the Two-End Black Coat Color Phenotype in Chinese Local Pigs
Source: Animals (Basel). 2025 Feb 7;15(4):478. doi: 10.3390/ani15040478 (PMC11851453; doi:10.3390/ani15040478)

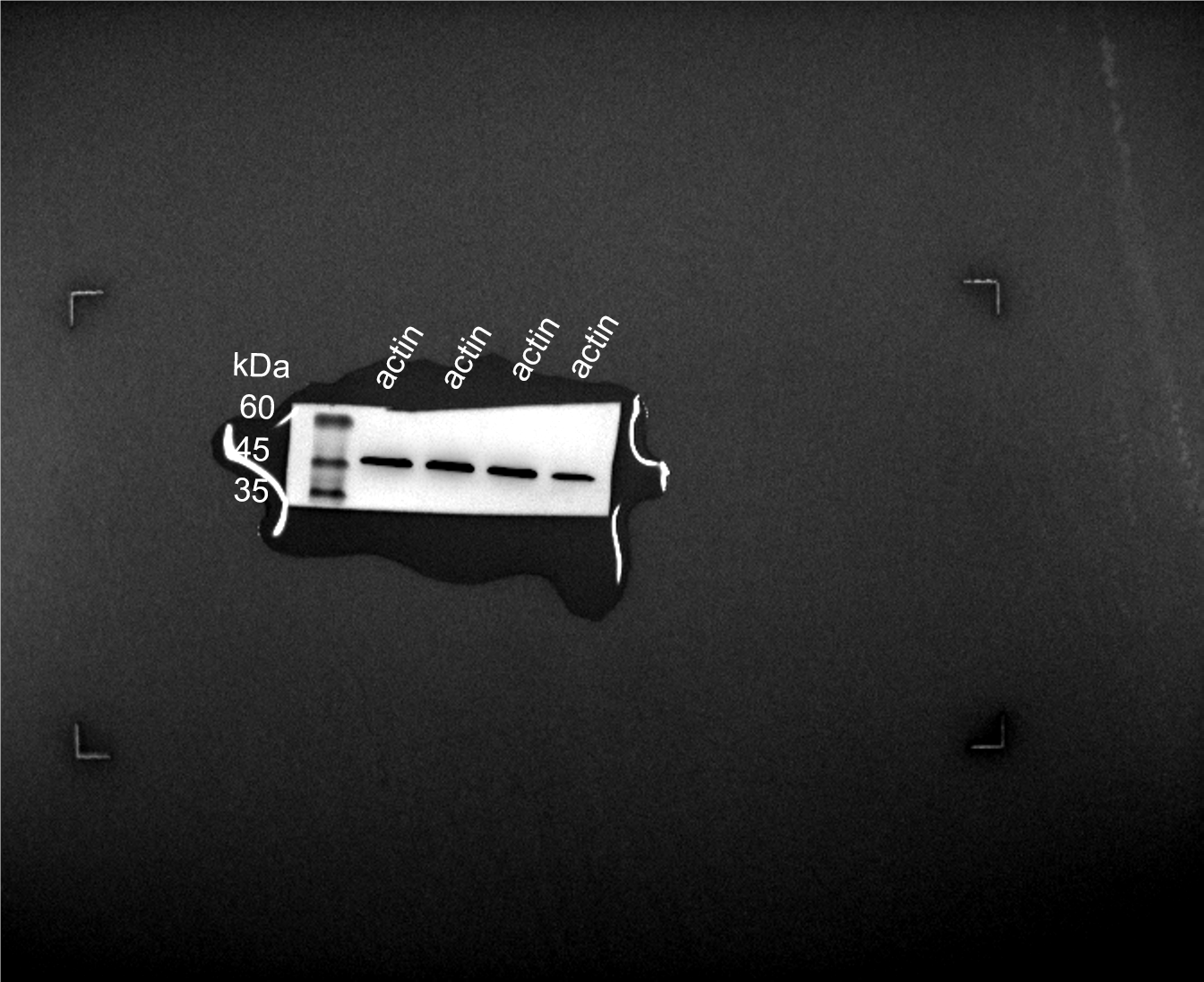

Supplement: Supplementary file 1 [file animals-15-00478-s001.zip › animals-3425682-supplementary/Original western blot figures/EDNRB-actin.tif]

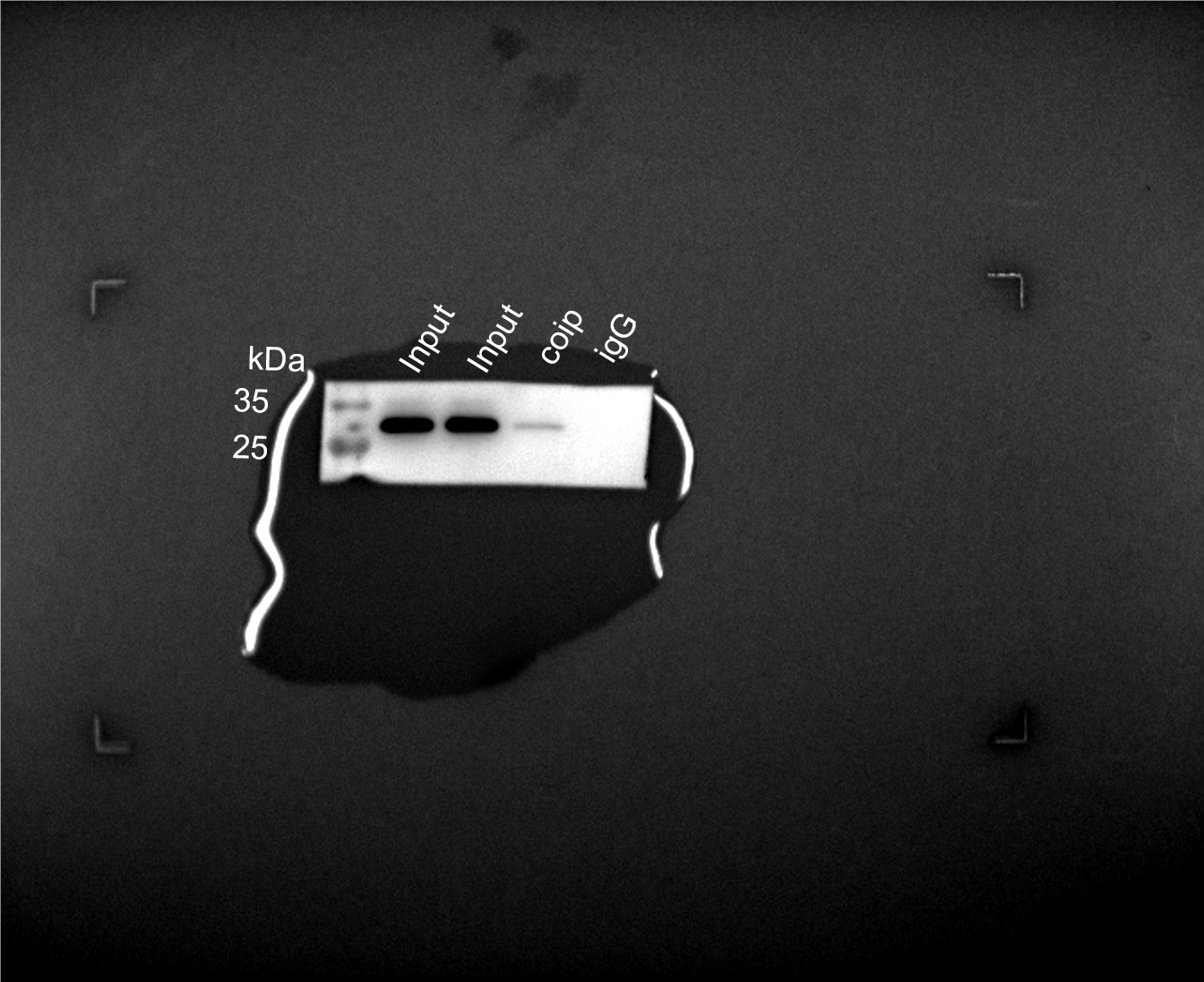

Supplement: Supplementary file 1 [file animals-15-00478-s001.zip › animals-3425682-supplementary/Original western blot figures/EDNRB-EDN1.tif]

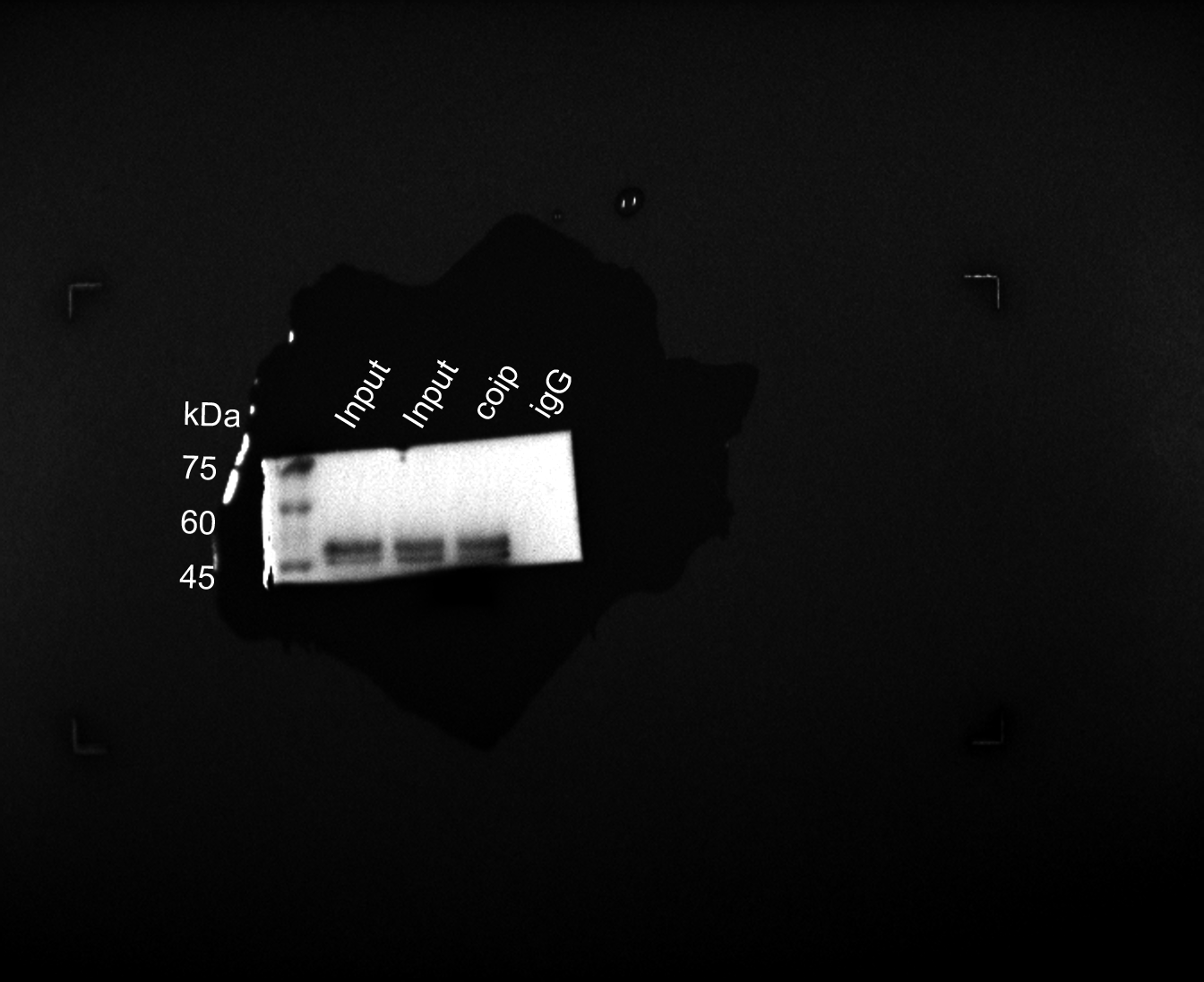

Supplement: Supplementary file 1 [file animals-15-00478-s001.zip › animals-3425682-supplementary/Original western blot figures/EDNRB.tif]

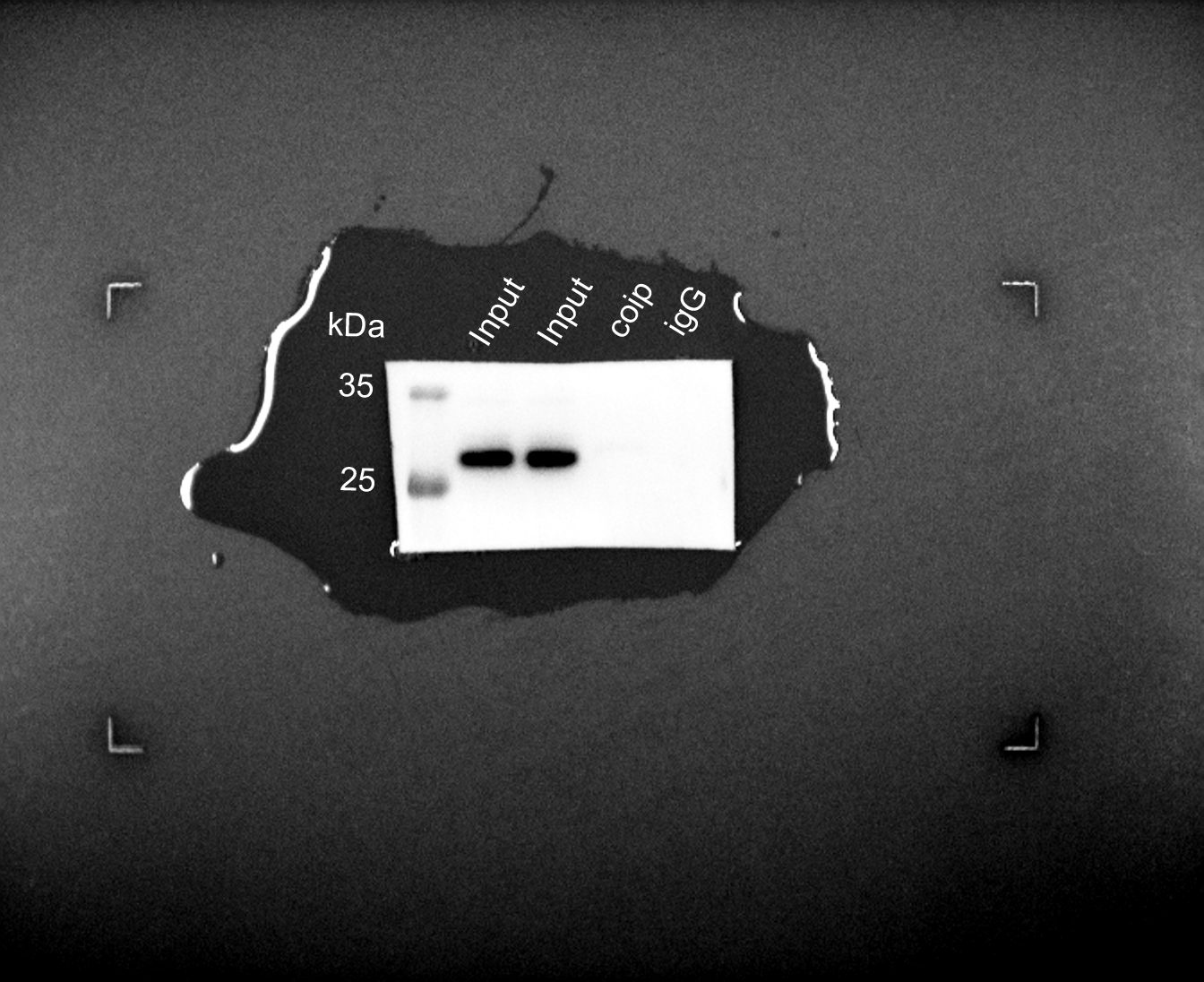

Supplement: Supplementary file 1 [file animals-15-00478-s001.zip › animals-3425682-supplementary/Original western blot figures/EDNRB_M -EDN1.tif]

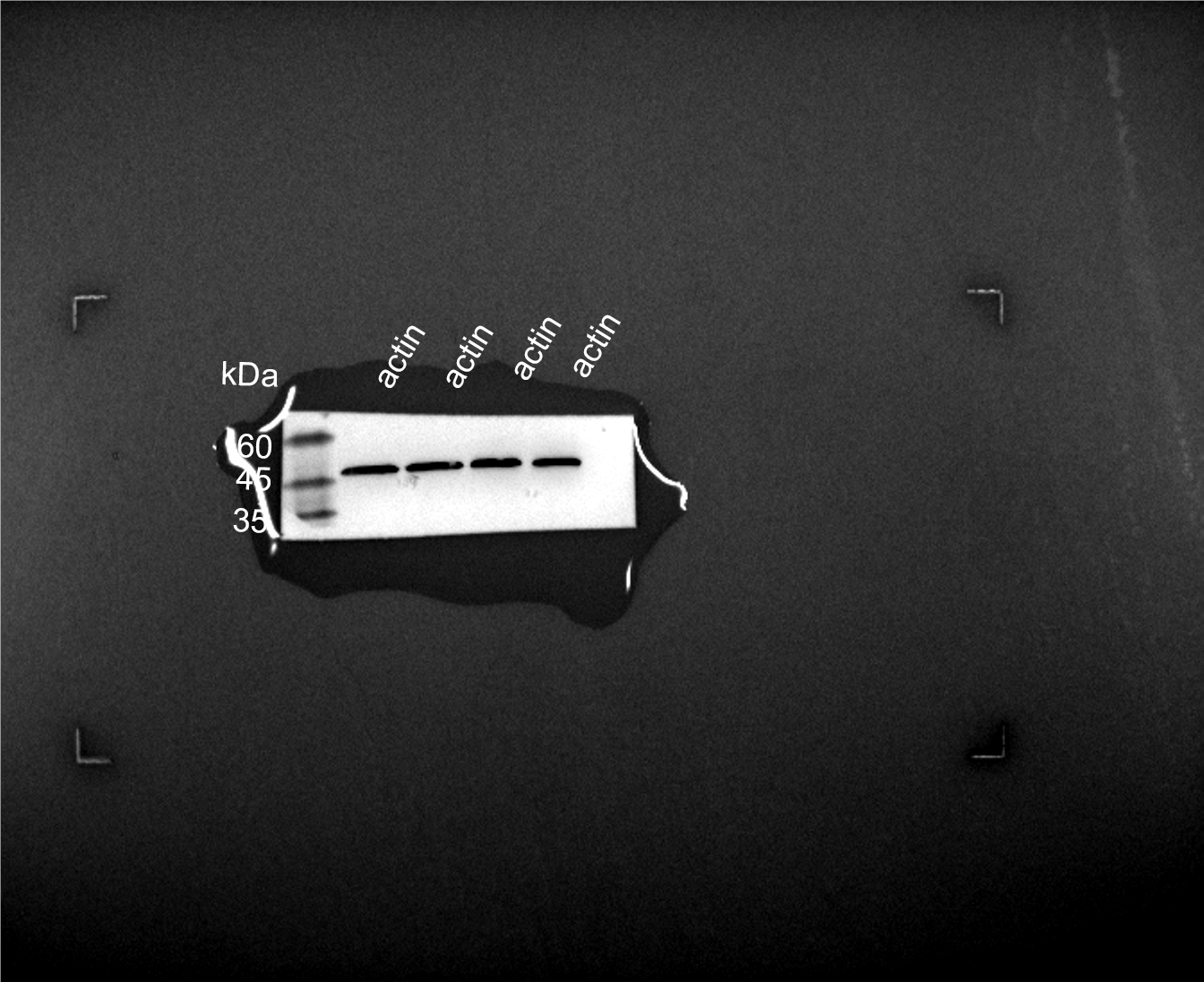

Supplement: Supplementary file 1 [file animals-15-00478-s001.zip › animals-3425682-supplementary/Original western blot figures/EDNRB_M-actin.tif]

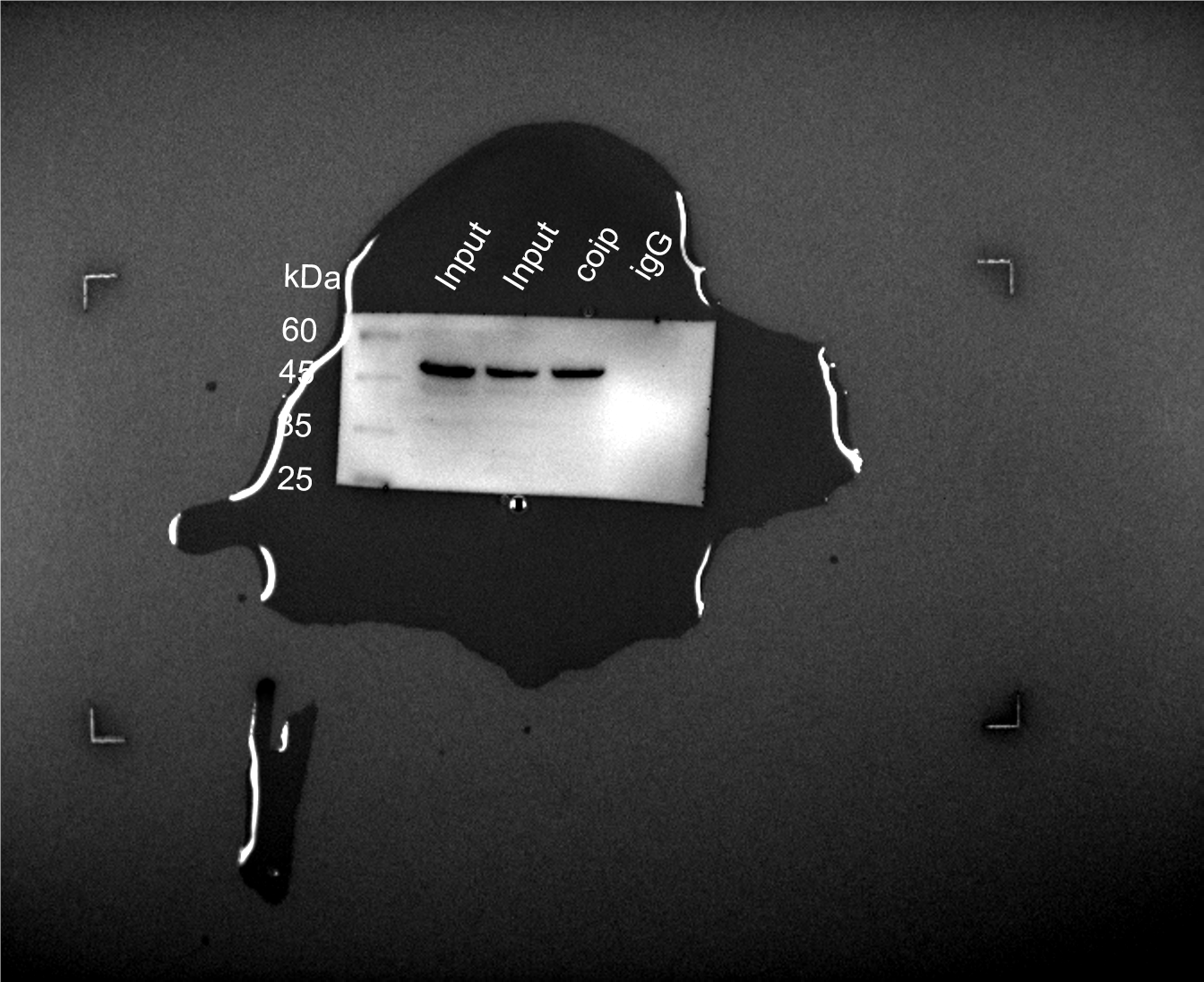

Supplement: Supplementary file 1 [file animals-15-00478-s001.zip › animals-3425682-supplementary/Original western blot figures/EDNRB_M.tif]
